# *Salmonella* sp. Tied to Multistate Outbreak Isolated from Wastewater, United States, 2022

**DOI:** 10.3201/eid3012.240443

**Published:** 2024-12

**Authors:** Zoe S. Goldblum, Nkuchia M. M’ikanatha, Erin M. Nawrocki, Nicholas Cesari, Jasna Kovac, Edward G. Dudley

**Affiliations:** The Pennsylvania State University, University Park, Pennsylvania, USA (Z.S. Goldblum, N.M. M’ikanatha, E.M. Nawrocki, J. Kovac, E.G. Dudley); Pennsylvania Department of Health, Harrisburg, Pennsylvania (N.M. M’ikanatha, N. Cesari)

**Keywords:** Salmonella enterica, bacteria, outbreak, wastewater, Senftenberg, wastewater surveillance, United States

## Abstract

We isolated *Salmonella enterica* serovar Senftenberg in raw wastewater from 2 Pennsylvania wastewater treatment facilities during June 2022. Whole genome sequencing revealed 4 isolates separated by <4 single nucleotide polymorphisms from *S*. *enterica* Senftenberg in a cluster from the 2022 nationwide outbreak linked to contaminated peanut butter.

The COVID-19 pandemic showcased the power of wastewater-based surveillance (WBS) to infer viral loads in communities (*1*). Health officials have also used WBS to track antimicrobial resistance (*2*,*3*), and some researchers have made associations between bacterial pathogens (e.g., *Salmonella enterica*) from domestic wastewater and those from clinical sources (*4*–*7*). The extent to which routine surveillance for bacterial pathogens would benefit public health is unclear. To evaluate possible benefits, we screened samples from 2 wastewater facilities for *S*. *enterica* during June 2022, concurrent with a study investigating SARS-CoV-2 in wastewater (*8*). Our study also included a broader investigation into whether we could isolate *S. enterica* that matched human isolates from ongoing outbreaks.

We collected composite wastewater samples twice a week during June 2022 from 2 wastewater (sewage) treatment facilities in central Pennsylvania, designated WWTP-1 and WWTP-2 (Table). We selected the 2 facilities based on their convenience and suitability: both were within a 1-hour driving radius from our laboratory and water treatment in both facilities focused solely on domestic sewage. WWTP-1 received wastewater from a population of ≈3,600 persons, and WWTP-2 received wastewater from a population of ≈13,600 persons. We adapted protocols from the US Food and Drug Administration’s Bacteriological Analytical Manual (https://www.fda.gov/food/laboratory-methods-food/bacteriological-analytical-manual-bam) to identify *S. enterica* isolates. In brief, we centrifuged 120 mL of wastewater at 5,000 *g* for 20 minutes at 4°C to precipitate solids. We then passed the supernatant through a 0.45-µm filter, and the filter was added to the pellet along with 40 mL of buffered peptone water supplemented with 20 mg/L of novobiocin. After a 24-hour recovery at 35°C, we subcultured the samples into selective media (tetrathionate and Rappaport-Vassiliadis broths) and incubated them at 42°C for 24 hours. We plated 10-µL aliquots of each enrichment culture on xylose lysine deoxycholate and Hektoen enteric agars and monitored them for growth and colonies characteristic of *Salmonella* sp. We restreaked putative *S*. *enterica* colonies to purify them and confirmed identity by PCR targeting *invA*. We constructed libraries by using the Nextera XT DNA Library Preparation Kit (Illumina, https://www.illumina.com) and used the MiSeq Reagent Kit v3 (Illumina) to perform sequencing at 500 (2 × 250) cycles. 

**Table:** Data for *Salmonella* sp. linked to multistate outbreak isolated from wastewater treatment facilities, United States, 2022*

Isolate no.	Isolation date, 2022	Location	Biosample accession no.
PSU-5375	June 15	WWTP-1	SAMN33902330
PSU-5376	June 15	WWTP-1	SAMN33902331
PSU-5387	June 22	WWTP-1	SAMN33902342
PSU-5398	June 16	WWTP-2	SAMN34154796

*WWTP-1, wastewater treatment plant 1; WWTP-2, wastewater treatment plant 2. Accession nos. from National Center for Biotechnology Information’s Sequence Read Archive (https://www.ncbi.nlm.nih.gov/sra).

We uploaded short sequence reads to the National Center for Biotechnology Information Pathogen Detection site (https://www.ncbi.nlm.nih.gov/pathogens) for genomic comparison to human clinical isolates and to identify molecular serovars. We deposited all sequencing data in the National Center for Biotechnology Information’s Sequence Read Archive (https://www.ncbi.nlm.nih.gov/sra) under BioProject PRJNA357723. We constructed an annotated phylogenetic tree by importing the Newick data from the National Center for Biotechnology Information’s Pathogen Detection database into the Interactive Tree of Life (https://itol.embl.de). We verified clusters on the Centers for Disease Control and Prevention’s System for Enteric Disease Response, Investigation, and Coordination (SEDRIC; https://www.cdc.gov/foodborne-outbreaks/php/foodsafety/tools/index.html).

During June 13–29, 2022, we isolated 42 *Salmonella* strains from wastewater samples. We conducted this *Salmonella* study alongside SARS-CoV-2 emergency response efforts, while new protocols were being validated, which affected its timing and duration. Whole-genome sequencing rendered isolates that we aligned to various serovars; Panama was the most prevalent (16 [38.1%]), followed by Senftenberg (9 [21.4%]) and Baildon (8 [19.0%]). Other serovars included Agona (3 [7.1%]), Oranienburg (3 [7.1%]), Montevideo (2 [4.8%]), and Kintambo (1 [2.4%]). We noted 4 serovar Senftenberg isolates were separated by 0–4 single nucleotide polymorphisms from a cluster of 40 clinical isolates uploaded to the Pathogen Detection database during March 2022–May 2023 (Table, Figure). Using SEDRIC, we verified that 21 (52.5%) of the clinical isolates matched a 2022 multistate foodborne outbreak of salmonellosis (designated cluster 2205MLJMP-1 in SEDRIC) that was sourced to contaminated peanut butter. Patients lived in 17 different states (https://www.cdc.gov/salmonella/senftenberg-05-22/map.html). Although Pennsylvania was not initially included, health officials eventually identified 3 additional Senftenberg isolates matching 2205MLJMP-1 in SEDRIC. Those isolates were from 3 Pennsylvania patients (PNUSAS320949, PNUSAS280385, PNUSAS280378 [Figure]), and researchers detected them after the outbreak investigation concluded on May 9, 2022. Researchers also connected 8 Baildon isolates to a previously documented outbreak of salmonellosis that occurred primarily in Pennsylvania (*9*).

**Figure:**
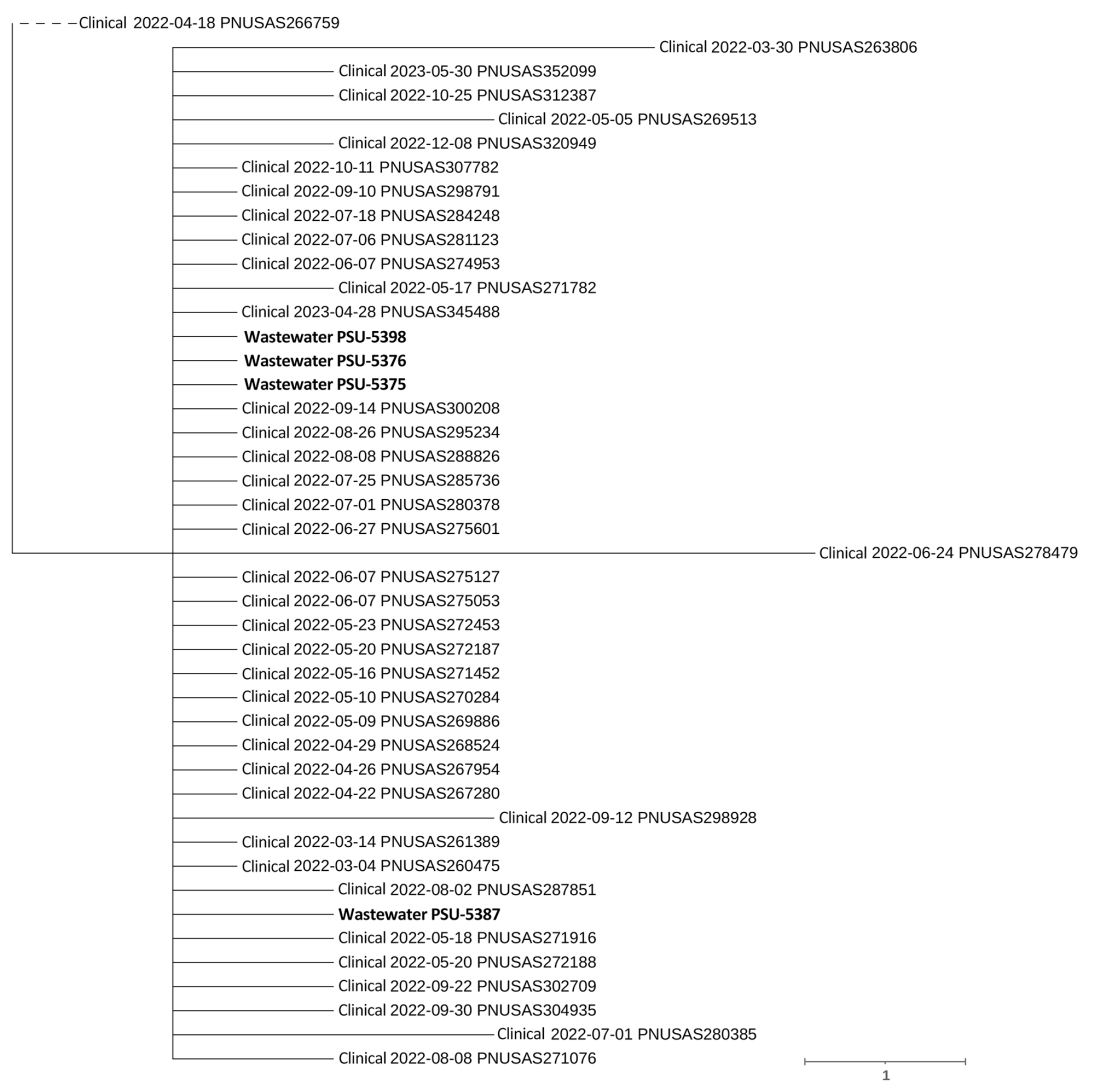
Isolates of *Salmonella* sp. linked to multistate outbreak isolated from wastewater treatment facilities, United States, 2022. We detected *S. enterica* serovar Senftenberg from 2 Pennsylvania wastewater facilities genetically linked to those associated with a 2022 multistate outbreak. SNP-based tree constructed using Newick data generated by the National Center for Biotechnology Information’s Pathogen Detection database (https://www.ncbi.nlm.nih.gov/pathogens), showing the relationship between 4 *S. enterica* Senftenberg isolates reported in this study (bold) and whole-genome sequence previously isolated from human cases within the same cluster. Note that dates indicate when data were uploaded to the pathogen detection database and are not necessarily the date of isolation. Scale bar indicates nucleotide substitutions per site. SNP, single-nucleotide polymorphism.

In conclusion, we isolated *S*. *enterica* Senftenberg from 2 rural wastewater treatment facilities in Pennsylvania and found the isolates to be genomic matches to strains associated with a multistate salmonellosis outbreak. No human cases of salmonellosis were reported in Pennsylvania at the time of that outbreak, possibly because of underreporting of nontyphoidal *Salmonella* (*10*). Our results highlight *Salmonella* strains reported to public health authorities that were not initially recognized as part of a multistate outbreak. We were only able to uncover this connection by comparing the genetic relatedness of *Salmonella* Senftenberg isolates from clinical and wastewater sources. The results of our study underscore the value of wastewater testing and targeted sewerage monitoring, not only in facilitating outbreak investigations but also in identifying additional cases, even after an outbreak has been declared closed. Our findings also highlight the need for further research into how targeted sewage monitoring can inform outbreak duration, prevention efforts, and regulatory oversight related to foodborne illnesses.
